# Upper gastrointestinal endoscopy in emergency setting for patients receiving oral anticoagulants – practice updates

**Published:** 2017

**Authors:** R Oprita, B Oprita, B Diaconescu, MR Bratu, D Berceanu

**Affiliations:** * ”Carol Davila” University of Medicine and Pharmacy, Bucharest, Romania; ** Clinical Emergency Hospital of Bucharest, Bucharest, Romania

**Keywords:** anticoagulant, endoscopy, bleeding, emergency, update

## Abstract

Anticoagulants are frequently used medications in diverse cardiovascular diseases. Their uses highly increase the risk of bleeding from upper and lower gastrointestinal sources, whether there is a classic vitamin K antagonist or a novel oral anticoagulant. Their interruption can promote procoagulation status with different thromboembolic accidents. Discontinuation of oral anticoagulants before the elective procedures is standardized but there are no guidelines for managing bleeding lesions of upper gastrointestinal tract concomitant with anticoagulation. Also, because some of the anticoagulants are new comers, there is no specific antidote, and so their anticoagulation effect cannot be antagonized fast in order to reduce the bleeding. Therefore, the endoscopic hemostasis must be definitive and efficient. This is a short review of the current management for the bleeding lesions of the upper gastrointestinal tract in patients taking oral anticoagulants.

## Introduction

Antithrombotic therapy is used to reduce the risk of thromboembolic events in patients with various pathologies, of which the most common are: atrial fibrillation, acute coronary syndrome, deep vein thrombosis, hypercoagulable states, and prosthetic valves. Antithrombotic drugs are classified into two major classes: antiplatelets and anticoagulants. Anticoagulants prevent clots by acting on blood coagulation cascade and are divided into 4 classes:

- Vitamin K antagonists (VKA) - acenocoumarol, warfarin;

 - Heparin and heparin derivatives - unfractionated heparin, fondaparinux, low molecular weight heparins (LMHW);

 - Direct factor Xa inhibitors - rivaroxaban (Xarelto), apixaban (Eliquis);

 - Direct thrombin inhibitors - dabigatran (Pradaxa).

Vitamin K antagonists are a family of drugs that provide competitive inhibition of vitamin K in the hepatocytes. Thus, it causes a reduction in the hepatic synthesis of coagulation factors whose production is dependent on vitamin K: prothrombin (II) with the longest half-life (72 hours), proconvertin (VII) with the shortest half-life (6 h), Stuart factor (X), antihemophilic factor B (IX), protein C and protein S. The indirect effect due to the inhibition of vitamin K explains the absence of the therapeutic effect as the drugs are initially introduced and the anticoagulation persists when the medication is stopped. VKA have pharmacodynamic characteristics are likely to lead to drug interactions: strong digestive absorption (interference with treatments influencing intestinal transit), blood transport with strong protein binding at a value of around 95% (likely to be modified by competing drugs), hepatic metabolism (with possible enzyme induction and inhibition).

The last two categories mentioned earlier are new-generation oral anticoagulant agents also known as direct oral anticoagulants (DOACs) used for the prevention of systemic embolism in non-rheumatic atrial fibrillation, as well as treatment and prevention of deep vein thrombosis or pulmonary thromboembolism. They are not recommended in patients with mechanical heart valves. These drugs offer certain advantages over vitamin K antagonists as they are prescribed at a daily fixed dose without the need to monitor the intensity of anticoagulation and the need to adjust the dosage accordingly. Furthermore, the interaction with other drugs is limited and the various foods do not alter the drug absorption. Also, the rapid onset of the anticoagulation effect as well as their short half-life facilitate the introduction and the interruption of these drugs. Currently, there is not an antidote for the clinical practice, but more substances are in the research stage with promising results (idarucizumab for dabigatran) [**1**]. All the new oral anticoagulant drugs are excreted to some extent through the kidney, but the elimination of the direct thrombin inhibitor, Dabigatran, is mostly influenced by the renal function. Compared to VKA, the new generation of oral anticoagulants has a lower risk of major bleeding events, particularly a significant reduction in the risk of intracerebral hemorrhage by 50%. Instead, dabigatran and rivaroxaban increase the risk of gastrointestinal bleeding (50% for dabigatran) by a reduced bioavailability, which increases the concentration of these drugs in the faeces and produces a local anticoagulant effect on the intestinal wall [**2**,**3**]. The predictive factors for an increased risk of bleeding are advanced age, renal dysfunction and the existence of a previous stroke.

**Fig. 1:**
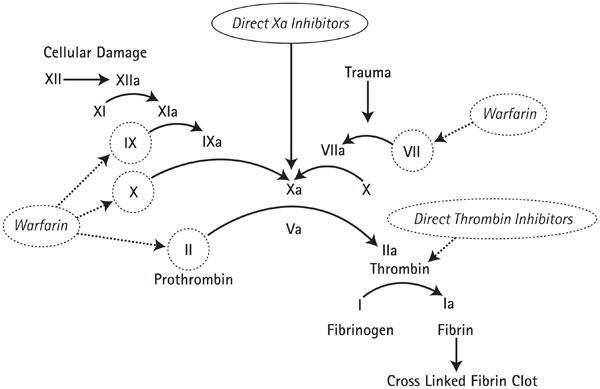
Mechanism of action of anticoagulants

Dabigatran peak plasma level is reached 3 hours after the administration of this drug, with a dose-independent half-life of 12-14 hours, prolonged in the case of renal dysfunction. It is contraindicated when the creatinine clearance is ≤ 30 ml/ min, which increases the half time to 27 hours. The recommended dose is 150 mg b.i.d., with a reduction to 110 mg b.i.d. if the patient is over 80 years or CrCl ≤ 50 mL/ min. Hepatotoxicity is not usually encountered as an adverse effect and dabigatran can be used in patients with hepatic impairment as long as an associated coagulopathy does not exist.

Gastrointestinal bleeding is encountered as an adverse effect of anticoagulation therapy, and its use is associated with a higher risk of bleeding after certain endoscopic procedures. Thus, for anticoagulated patients requiring endoscopy, the following factors should be taken into account before the procedure:

- Elective vs. emergency endoscopy;

- The bleeding risk of the procedure;

- The effect of antithrombotic therapy on the risk of bleeding;

- Risk of thromboembolic events associated with the discontinuation of periprocedural anticoagulation.

Acute GI bleeding emerging in patients under anticoagulation therapy raises several problems regarding the balance between drug interruptions and the continuation of the therapy. Gastroenterologists who take care of these patients have largely varying attitudes and an overall limited knowledge of this topic. This is related to the scarcity of the studies focusing on the issue of acute GI bleeding in anticoagulated patients and the absence of randomized controlled trials analyzing the different approaches. Furthermore, practice guidelines by GI professional societies only marginally address this topic as they mostly focus on the management of anticoagulants in patients undergoing elective procedures. This absence of data is even more relevant for DOACs.

The bleeding risk varies from one elective endoscopic procedure to another. The following table summarizes the main elective endoscopic procedures that are classified based on the risk of bleeding.

**Table 1:** Main elective endoscopic procedures classified based on the risk of bleeding

High risk procedures	Low risk procedures
Polypectomy	Diagnostic ***upper gastrointestinal (UGI) endoscopy*** or colonoscopy, including mucosal biopsy
Pancreatic or biliary sphincterotomy	Endoscopic retrograde cholangiopancreatography with stent implantation (biliary or pancreatic) or papillary balloon dilation without sphincterotomy
Variceal ligation	Diagnostic endoscopy
Therapeutic endoscopy	Endoscopic capsule
PEG (percutaneous endoscopic gastrostomy) installation or PEJ	EUS (endoscopic ultrasound) without FNA (fine needle aspiration) biopsy
Endoscopic hemostasis	Argon Plasma Coagulation
Tumor ablation	Endoscopic ablation of Barrett’s esophagus
EUS with fine needle aspiration biopsy (FNAB)	
Ampullary resection	
Endoscopic mucosal resection	
Endoscopic submucosal dissection	
Balloon or bougie dilation	

A gastrointestinal bleeding, which occurs when the patient receives antithrombotics, is a therapeutic challenge. The clinician is faced with the decision to stop the anticoagulation, with thromboembolic consequences deriving from it, or to continue the anticoagulation with the risk of exsanguination. Despite the progress in monitoring the anticoagulated patients, bleeding complications are common and a severe bleeding episode is recorded in 20% of the cases. Most studies showed that the source of bleeding is mucosal in the majority of cases of patients taking anticoagulants, endoscopy being therefore mandatory for this category of patients. No significant differences were recorded regarding the etiology or the location of the gastrointestinal bleeding between the general population and those receiving oral anticoagulants.

The risk of gastrointestinal bleeding in patients taking VKA can be appreciated by using the HAS-BLED score:

**Fig. 2:**
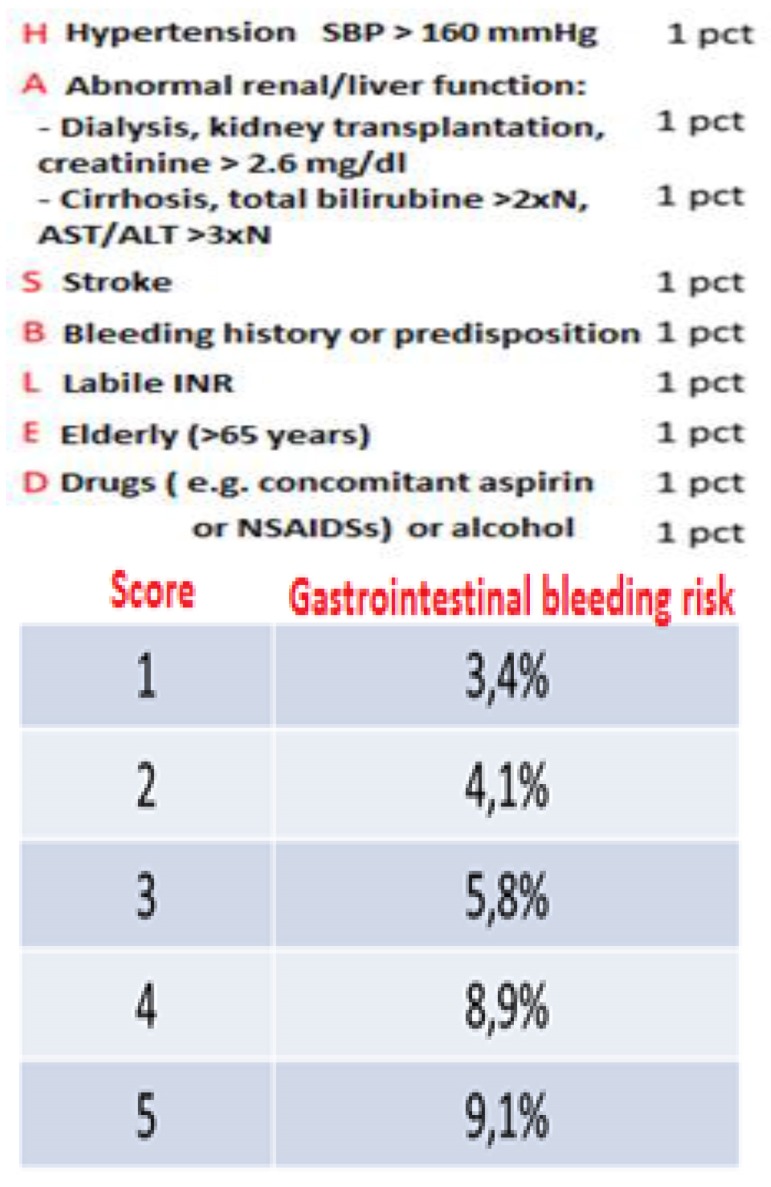
HAS-BLED score

*Note: A score higher than 6 was too rare to determine the risk for GI bleeding. Also, the HAS-BLED score was validated for warfarin, but not for the new anticoagulants.

In patients treated with Acenocumarol, the risk of bleeding is relatively small compared to the general population; the highest risk of bleeding being encountered within the first month of treatment. A value of INR between 1.2 and 1.5 creates a risk similar to that in the general population. The risk increases dramatically when the INR > 4.5.

In a retrospective study of 52 patients, the correction of the INR value in the range of [1.5; 2.5] enabled the diagnostic and the therapeutic endoscopic intervention with a success rate comparable to that achieved in patients who were not receiving anticoagulants [**4**]. A source of bleeding was found in 83% of the cases, a percentage slightly lower than the control-group (92%). The duration or the intensity of anticoagulation were not predictors in terms of the diagnosis rate. It was believed that the source of bleeding was most frequently identified when the GI bleeding was associated with a value of INR ≤ 3 [**5**]. Permanent hemostasis was achieved in 91% of the cases without any complications related to the endoscopic intervention, and the most common cause identified was peptic ulcer disease.

In a larger study in which 95% of the patients had an INR between 1.3 and 2.7, the endoscopic hemostasis had an initial success rate of 95% (233/ 246 patients) using methods such as application of endoscopic hemoclips, adrenaline injection, or thermocoagulation. Although the rate of rebleeding was 23%, the INR before endoscopic treatment was not correlated with the risk of subsequent rebleeding, the need for surgery, transfusion therapy, duration of hospitalization or mortality. A mild or moderate anticoagulation intensity does not increase the risk of rebleeding after an endoscopic treatment for non-variceal gastrointestinal bleeding, which suggests that endoscopy is appropriate and safe to practice in these patients [**6**]. The correction of coagulopathy should not delay the endoscopic intervention.

Another retrospective study showed that the rate of rebleeding in patients with supratherapeutic INR (INR ≥ 4) did not differ substantially from the rebleeding rate in patients with INR in the therapeutic range (2.0-3.9) [**7**]. Finally, a meta-analysis on 1869 patients who presented with non-variceal upper gastrointestinal bleeding, the INR value at presentation was not correlated with the risk of rebleeding [**8**]. Instead, an INR ≥ 1.5 was associated with a higher mortality rate.

Given the results of the above studies, the upper gastrointestinal endoscopy with endoscopic hemostasis is very effective even in patients with a moderately increased INR. The INR normalization did not reduce the risk of rebleeding and only delayed the endoscopic intervention. In case of massive bleeding, an INR less than 2.5 is considered reasonable for practicing emergency hemostasis within safety limits.

The decision to stop, reduce the dose, or antagonize the anticoagulation therapy must be carefully weighed against the risk of an ongoing bleeding and the development of hemorrhagic shock. In most cases, the anticoagulant therapy is interrupted to facilitate the endoscopic hemostasis. The American College of Clinical Pharmacology (ACCP) recommend the stopping of the therapy with VKAs and the antagonizing of the anticoagulation with prothrombin complex concentrate (PCC) and not fresh frozen plasma (FFP), in case of major bleeding. Also, it is advised to use vitamin K (5-10 mg iv slowly) associated with PCC [**9**]. Prothrombin complex concentrate contains vitamin K dependent clotting factors (II, VII, IX, X) and is found in inactive or partially activated forms. The 2014 guidelines of the American Society of Cardiology for the management of patients with mechanical heart valves recommend FFP for those with incontrollable bleeding for the reversal of anticoagulation. Vitamin K is not recommended because it usually induces a hypercoagulable state [**10**].

Regarding the new generation of anticoagulants, since there are no clinical studies to determine the best therapeutic conduct in case of a gastrointestinal bleeding, the current recommendations are based on the experts’ opinions. The initial management of the bleeding episode depends on its severity.

When the hemorrhage is not severe, the mere interruption of the anticoagulant may be sufficient. Severe GI bleeding requires emergency gestures such as hydroelectrolytic correction, blood transfusions to correct anemia (Hb ≤ 7 g/ dl), etc. Also, the aggressive fluid resuscitation is essential in order to normalize the blood urea nitrogen (BUN) level for dabigatran because it promotes the renal excretion of the drug. It is important to find out when the last dose was administrated and the half-life can be found by measuring serum creatinine and by calculating the creatinine clearance. The dabigatran concentration can be determined by using the Hemoclot test, and the anti-factor Xa activity can be monitored for apixaban and rivaroxaban [**11**]. Protamine sulfate and vitamin K have no effect on the direct oral anticoagulants. Anti-fibrinolytics effectiveness such as tranexamic acid is not known but its use is reasonable in some selected patients. Similarly, the effect of desmopressin, which is independent of thrombin or factor Xa, appears to be beneficial but requires further studies [**12**].

In case of a massive bleeding, hemodialysis can be used for dabigatran but not for rivaroxaban or apixaban, because they have a lower renal excretion rate and a much stronger protein binding. When the bleeding is caused by DOACs, the efficiency of fresh frozen plasma is uncertain [**13**]. The prothrombin complex concentrate as well as rVIIa were not included in the clinical studies that observed patients with GI hemorrhage. Instead, in healthy individuals, the altered coagulation tests modified by rivaroxaban are corrected by the administration of FFP, but not in the case of dabigatran [**14**]. This result does not indicate that the FFP would have any clear benefits in patients with gastrointestinal bleeding. In patients with life threatening bleeding it is recommended to administer FFP 40-50 IU/ kg but with there is no clear evidence of any benefit [**15**]. The opinion of a surgeon and interventional radiologist is mandatory in case of a massive bleeding that cannot be controlled otherwise.

The endoscopic procedures used to control a gastrointestinal bleeding are: injection of epinephrine 1: 10.000 in the submucosa, Argon plasma coagulation (easy to use, but with an increased risk of cecal perforation), monopolar or bipolar electrocoagulation (used to treat vascular lesions-Gold Probe), mechanical hemostasis (used to enhance the hemostasis achieved with bipolar electrocoagulation), sclerotherapy (rarely used). For anticoagulated patients, electrocoagulation has its risks: tissue damage and rebleeding. The method of choice is the mechanical hemostasis with clips.

Generally, the anticoagulant therapy can be resumed after 4 days of effective hemostasis. Intravenous heparin can be used for the high-risk patients requiring permanent anticoagulation. The decision to restart the antithrombotics should be evaluated from one patient to another; the early resumption of anticoagulation is recommended (earlier than 7 days) in patients with an increased risk of thromboembolic events. Usually, vitamin K antagonists are reintroduced 7-15 days after the acute event.

The figure below illustrates some endoscopic lesions found in patients who were anticoagulated at the time of the endoscopy (note the frequently used endoscopic hemoclips):

**Fig. 3:**
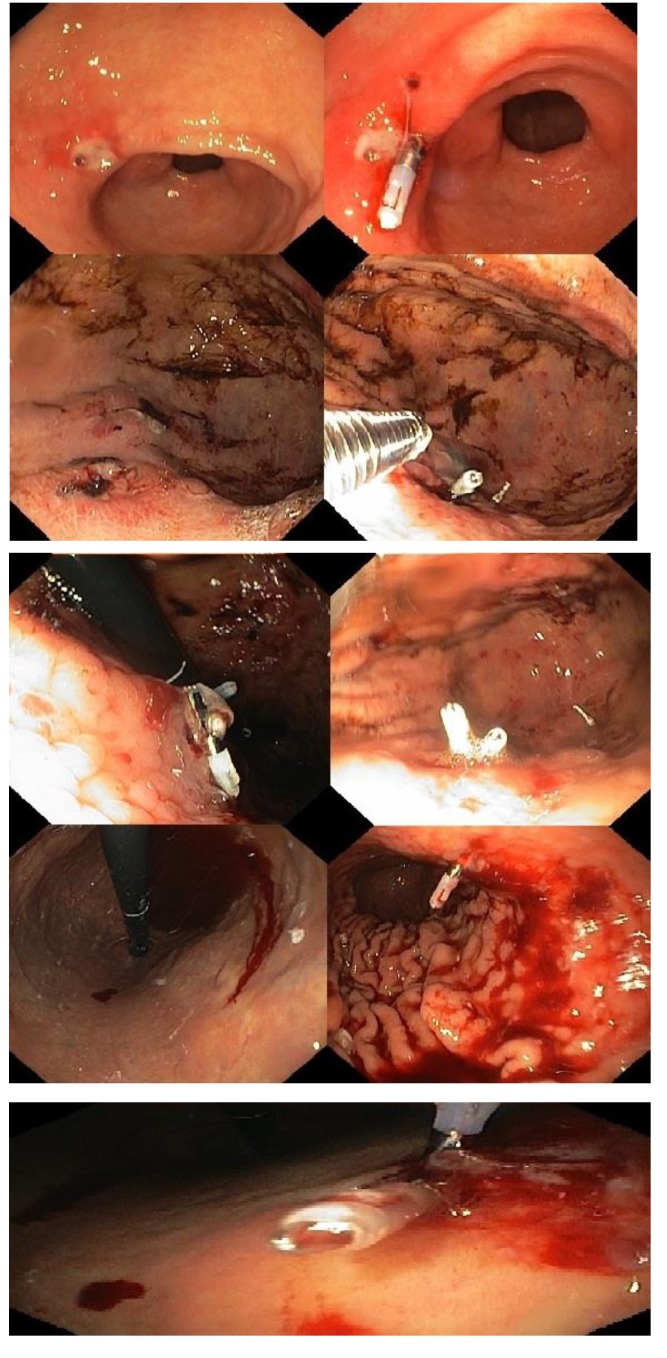
Endoscopic lesions found in patients who were anticoagulated at the time of the endoscopy

**Table 2:** Anticoagulant and half-life cases and their emergency management

Anticoagulant	Half-life	Emergency management
VKAs (Acenocumarol)	8-11 h	1. Vit K (i.v./ p.o.) 1-10mg
		2. FFP 10-30ml/ kg
		3. Prothrombin complex concentrate 25-50 U/ kg i.v.
Unfractionated heparin	IV: 1h	Protamine sulfate – pay attention to hypotension and anaphylaxis
	SC: 2-4 h	
LMWH	IV: 4 h	1. Protamine sulfate
	SC: 12-20 h	2. rVIIa
Fondaparinux		1. Protamine sulfate
		2. rVIIa
Apixaban	8-13 h	1. Supportive care
		2. Activated charcoal (if the last dose was administered 2-3 h before);
		3. Factor VIIa
		4. Prothrombin complex concentrate 25-50 U/ kg i.v.
Rivaroxaban	5-9 h	5. Desmopressin
		6. Antifibrinolytics
Dabigatran	13-27 h	1. Activated charcoal (if the last dose was administered 2-3 h before)
		2. Prothrombin complex concentrate
		3. Factor VIIa
		4. Hemodialysis

**Acknowledgements**

We would like to thank the whole emergency department team and the endoscopy lab team for supporting our protocols.

**Sources of Funding**

Personal.

**Disclosures**

None.

## References

[1] Lu G, DeGuzman  FR, Hollenbach  SJ, Karbarz  MJ, Abe  K, Lee  G (2013). A specific antidote for reversal of anticoagulation by direct and indirect inhibitors of coagulation factor Xa. Nat Med.

[2] Connolly SJ, Ezekowitz MD, Yusuf S (2009). RE-LY Steering Committee and Investigators. Dabigatran versus warfarin in patients with atrial fibrillation. N Engl J Med.

[3] Blech S, Ebner T, Ludwig-Schwellinger E (2008). The metabolism and disposition of the oral direct thrombin inhibitor, dabigatran, in humans. Drug Metabolsim and Disposition.

[4] Choudari CP, Rajgopal C, Palmer KR (1994). Acute gastrointestinal haemorrhage in anticoagulated patients: diagnoses and response to endoscopic treatment. Gut.

[5] Landefeld CS, Rosenblatt MW, Goldman L (1989). Bleeding in outpatients treated with warfarin: relation to the prothrombin time and important remediable lesions. Am J Med.

[6] Wolf AT, Wasan SK, Saltzman JR (2007). Impact of anticoagulation on rebleeding following endoscopic therapy for nonvariceal upper gastrointestinal hemorrhage. Am J Gastroenterol.

[7] Rubin TA, Murdoch M, Nelson DB (2008). Acute GI bleeding in the setting of supratherapeutic international normalized ratio in patients taking warfarin: endoscopic diagnosis, clinical management, and outcomes. Gastrointest Endosc.

[8] Shingina A, Barkun AN, Razzaghi A (2011). Systematic review: the presenting international normalized ratio (INR) as a predictor of outcome in patients with upper nonvariceal gastrointestinal bleeding. Aliment Pharmacol Ther.

[9] Holbrook A, Schulman  S, Witt  DM (2012). Evidence-Based Management of Anticoagulant Therapy: Antithrombotic Therapy and Prevention of Thrombosis, 9th ed: American College of Chest Physicians Evidence-Based Clinical Practice Guidelines. Chest.

[10] Nishimura  RA, Otto  CM, Bonow  RO (2014). 2014 AHA/ ACC guideline for the management of patients with valvular heart disease: a report of the American College of Cardiology/ American Heart Association Task Force on Practice Guidelines. Circulation.

[11] Stangier  J, Feuring  M (2012). Using the HEMOCLOT direct thrombin inhibitor assay to determine plasma concentrations of dabigatran. Blood Coagul Fibrinolysis.

[12] Siegal DM, Crowther  MA (2013). Acute management of bleeding in patients on novel oral anticoagulants. Eur Heart J.

[13] Kaatz S, Kouides  PA, Garcia DA (2012). Guidance on the emergent reversal of oral thrombin and factor Xa inhibitors. Am J Hematol.

[14] Eerenberg  ES, Kamphuisen PW, Sijpkens MK (2011). Reversal of rivaroxaban and dabigatran by prothrombin complex concentrate: a randomized, placebo-controlled, crossover study in healthy subjects. Circulation.

[15] Weitz  JI, Quinlan  DJ, Eikelboom  JW (2012). Periprocedural management and approach to bleeding in patients taking dabigatran. Circulation.

